# Three-dimensional surgical simulation for facial asymmetry: soft tissue-, skeleton-, and occlusion-based planning

**DOI:** 10.1186/s40902-017-0135-z

**Published:** 2017-12-05

**Authors:** Tae-Geon Kwon

**Affiliations:** Department of Oral and Maxillofacial Surgery, School of Dentistry, Kyungpook National University, Samduck 2 Ga, Jung Gu, Daegu, 700-421 South Korea

Using three-dimensional (3D) computerized simulation systems has enabled us to determine the anticipated position of the maxilla and mandibular proximal and distal segments. The bony segments need to be mobilized according to the planned final position. The concept of 3D surgical planning in orthognathic surgery has evolved continuously. While using 3D simulation for planning the correction of facial asymmetry, surgeons must consider how and where the bony segments can be positioned in 3D virtual space. The three different components of 3D surgical simulation for planning of dentofacial asymmetry correction are (1) soft tissue-, (2) skeleton-, and (3) occlusion-based segment mobilization.

## Soft tissue-based segment mobilization

The ultimate goal of facial asymmetry correction is creating a symmetrical soft tissue outline. Therefore, the osseous skeletal segments need to be mobilized to maximize soft tissue symmetry. There are many innovative ideas for the optimization of soft tissue simulation [1, 2]. However, even with great advancements in commercially available 3D planning software, there are still some limitations in perfect prediction [3, 4]. The soft tissue response after surgery can be influenced by the different characteristics of the skin, muscle, and fat tissues, all of which have different resilience levels [5, 6]. Therefore, the changes in the position of the underlying bones would not be directly translated to changes in the overlying soft tissue [7, 8].

## Skeleton-based segment mobilization

Skeleton-based simulation refers to the alignment of the skeleton to achieve a symmetrical mandibular/maxillary position, followed by the application of appropriate orthodontic movements according to this alignment. This implies that skeletal-based surgical planning could aggravate the occlusal interference or result in postoperative occlusal discrepancy. Thus, this planning should focus on maintaining the skeletal symmetry of the mandibular body, ramus, and chin areas [9, 10]. Another purpose of skeletal-based segment mobilization is to minimize the inter-bony interference in mandibular proximal/distal segments. If maxillary surgery is included and a yaw movement of the maxilla is applied, the distal segment of the mandible can be moved to minimize the inter-segment interference [11]. However, the degree of occlusal discrepancy that can be corrected by postoperative orthodontic treatment cannot be quantified. Moreover, there are limitations in estimating postoperative orthodontic movements before surgery. This idea can be used in most cases of 3D planning before initiating orthognathic treatment, especially for “surgery first” cases or for establishing the initial surgical treatment objectives (STOs).

## Occlusion-based segment mobilization

Surgical simulation based on changes in pre- and predicted postsurgical occlusion can be defined as “occlusion-based” segment mobilization [12]. This concept represents the 3D repositioning of skeletal segments without including orthodontic movements in computerized simulations, especially in cases of only mandibular surgery. For example, while performing the bilateral sagittal split ramus osteotomy for correcting asymmetric mandibular prognathism after using classical presurgical orthodontics, occlusion-based simulation would be helpful for simulating postoperative outcomes. Based on the simulated postoperative occlusion and intercuspation, distal mandibular segments would be mobilized. However, these movements cannot guarantee optimized skeletal symmetry [13]. Therefore, additional contouring surgery would be required to achieve optimal skeletal symmetry in the mandible.

In summary, the system for soft tissue-based surgical simulation needs improvement to reflect individual variation and differences in soft tissue characteristics. Skeletal-based segment mobilization can readily be used for initial STOs, surgery first approaches, or two-jaw surgery cases. Occlusion-based segment mobilization can be used for final STOs or mandibular surgery-only cases in which there will be limited orthodontic movement after orthognathic surgery.

Computerized 3D surgical simulation improves not only the accuracy of the surgical treatment of facial asymmetry, but also the esthetic outcomes. These computerized systems improve the overall system of surgical planning, the surgery itself, and the evaluation process. The proper application of adequate computerized 3D simulation concepts for orthognathic surgery is essential for successful results.
